# A Pronoun Analysis of Couples’ Support Transactions

**DOI:** 10.3389/fpsyg.2016.00077

**Published:** 2016-02-02

**Authors:** Céline Hinnekens, Gilbert Lemmens, Gaëlle Vanhee, Lesley Verhofstadt

**Affiliations:** ^1^Experimental-Clinical and Health Psychology, Ghent UniversityGhent, Belgium; ^2^Psychiatry and Medical Psychology, Ghent University HospitalGhent, Belgium

**Keywords:** close relationships, we-ness, support interactions, pronoun analyses, relationship satisfaction

## Abstract

The present study collected data about couples’ level of relationship quality and their usage of pronouns that express we-ness or separateness in the context of support interactions. The sample consisted of 48 couples in a long-term relationship who provided questionnaire data and participated in two videotaped social support interaction tasks. Couples’ videotaped interactions were subsequently coded for the number of personal pronouns—we-words (e.g., we, ours, ourselves) versus you and me-words (e.g., me, mine, you, yours)—used by both partners.

## Introduction

After their first individual efforts of coping with stressful events, people most likely turn to their intimate partner for support (Sullivan and Davila, 2010). Effective support provision in couples refers to partners being responsive to each other’s needs and includes all actions that express care, confirm the support recipient’s self-esteem, feelings or behaviors, and that support the partner in coping with the stressful event (e.g., practical assistance, providing information; Cutrona, 1996; Pasch and Bradbury, 1998; Rafaeli and Gleason, 2009). Empirical studies convincingly produced evidence for this kind of effective support being linked to relationship satisfaction and changes in relationship satisfaction (e.g., Saitzyk et al., 1997; Rafaeli and Gleason, 2009; Verhofstadt et al., 2013). An important mechanism assumed to underlie the connection between spousal support and relationship satisfaction concerns the presence of relationship schemas related to partners’ so-called sense of ‘we-ness.’ According to Cutrona (1996) spouses’ supportive actions not only reduce the immediate distress of a stressful event but they also foster the belief that the relationship may be an available supportive resource in times of adversity/hardship. This belief then influences the recipient’s evaluation of the quality of the relationship and satisfaction with the relationship (Sullivan and Davila, 2010). Bodenmann (2005) defines this sense of we-ness as partners’ belief that they are both committed to support each other in coping with personal difficulties. As such, a feeling of we-ness during support interactions reflects a common–as opposed to individual–experience of coping with stressful life events (Iafrate et al., 2012). Other authors, particularly known from the cognitive interdependence literature, define the concept of we-ness more broadly, as partners’ shared identity as a couple–as opposed to an identity as separate individuals (Agnew et al., 1998), whereas couples’ sense of separateness rather refers to an autonomous and individualistic representation of the self (an identity as separate individual).

Traditionally, measurements of partners’ sense of we-ness rely on self-report questionnaires (e.g., Inclusion of the Other in the Self Scale; Aron et al., 1992) or thematic/content analyses of partners’ descriptions of memories of events within the relationship (Krokoff et al., 1989) or the relationship itself (Fletcher et al., 1987). A third type of measurement draws from psycholinguistic research, and involves analyses of couples’ pronoun usage (e.g., Slatcher et al., 2008). Seider et al. (2009, p. 605) describe partners’ usage of first-person plural pronouns (e.g., we, us, our) versus the use of first- (e.g., I, me, my) or second-person singular (e.g., you, your) pronouns as a ‘reliable linguistic marker of an underlying shared versus separate dimension of identification.’ Other studies confirmed these findings and thus couples’ usage of we-words versus you/me-words may be considered to be an implicit but reliable measure of their sense of we-ness versus separateness (e.g., Reid et al., 2007; Rohrbaugh et al., 2012).

Pronoun analysis might be a particularly interesting approach to measure partners’ sense of we-ness –as a shared meaning structure– during support interactions as it is less biased than traditional self-report measures (Schwarz et al., 1998). Also couples’ pronoun usage is less controlled and suppressed than the content of their conversations or the behavior they display (Seider et al., 2009). Several studies found a beneficial role of not using first-person language use in dealing with stressful events (Kross et al., 2014). The use of non-first-person language during introspection was associated with self-distancing and this is in turn associated with less perceived distress (Kross et al., 2014; Park et al., 2015). Furthermore, a manipulation of the pronoun usage ‘we’ may lead to an increase in perceived relationship closeness and quality (Fitzsimons and Kay, 2004).

The aim of the current dataset was to complement existing pronoun research as well as research on support in couples by providing a pronoun analysis of partners’ sense of we-ness versus separateness during support interactions.

## Materials and Methods

### Ethics Statement

The study was approved by the ethical committee of the Faculty of Psychology and Educational Sciences of Ghent University, Belgium.

### Participants

Data were collected between July 2009 and January 2010. The sample consisted of 96 members of 48 Flemish heterosexual couples. The participants were recruited by a team of research assistants from the geographic vicinity of our research center. The inclusion criteria stipulated having a heterosexual relationship for at least 1 year and to be married/cohabiting. The mean ages for the men and the women were 41.70 years (*SD* = 14.65, range = 22–76), and 40.26 years (*SD* = 15.29, range = 20–77), respectively. At the time of the investigation, the average length of the couples’ cohabitation/marriage was 16.98 years (*SD* = 14.52, range = 1–55). After providing their written informed consent, both partners independently completed some online questionnaires (data on demographics and relationship functioning). Couples who completed the questionnaires were then scheduled to attend a laboratory session. In this session, the members of each couple participated in two 10-min videotaped support interaction tasks. At the end of the lab session, each couple was fully debriefed and received a gift voucher of 20 euros.

### Materials

#### Dyadic Adjustment Scale

The DAS is a widely used questionnaire to assess partners’ relationship satisfaction (DAS, Spanier, 1976). The mean values of the global DAS within this study were 113.16 for men and 115.52 for women (α = 0.89 and 0.90, respectively). DAS norms (Spanier, 1976) indicate an average satisfaction score of 114/115 for a married sample, thereby suggesting that our sample is comparable to an average group of married couples in terms of relationship satisfaction.

#### The Social Support Interaction Task

We applied a support interaction task similar to those used in previous observational research on spousal support (e.g., Pasch and Bradbury, 1998; Verhofstadt et al., 2008). The couples were guided into a laboratory that was furnished as a living room and was equipped so that their support interactions could be videotaped with their prior knowledge and consent. One spouse was designated to be the support seeker and the other spouse to be the support provider. For a random half of the couples in the first lab discussion, the male partner was designated as the support seeker, with the female partner in the role of the support provider. For the other half of the couples in the first discussion, these roles were reversed. In the second lab discussion, the partners traded their roles so that data could be obtained for both partners in both roles. Before each interaction, the designated support seeker was asked to discuss a salient personal problem (defined as any problem of which the source was not the partner or the relationship, such as dealing with work stress, tensions with family members, health issues) with his/her partner. The partners were allowed to interact up to a maximum time limit of 10 min.

#### Pronoun Analysis

In order to derive an index of participants’ sense of we-ness/separateness, a pronoun analysis was conducted on the collected observational data. The pronoun analysis was conducted by two trained research assistants and in accordance with the coding procedure developed by Seider et al. (2009; permission to use this procedure was obtained). The first step of the coding process consisted of identifying each pronoun used by our study participants. This required the verbatim transcription of the videotaped support interactions. Secondly, each pronoun was classified in one of three categories: (a) me-words, pronouns that refer to the self (e.g., me, my, mine, myself); (b) you-words, pronouns that refer to the partner (e.g., you, yours, yourself) and (c) we-words, pronouns that refer to the couple (e.g., we, ours, ourselves). This classification was based on the coding-dictionary (in Dutch) that is enclosed in the data-set accompanying the current manuscript (see below). Similar to Seider et al. (2009) the verbal context of participants’ pronoun was taken into account as well, given its influence on the meaning of a particular pronoun. A subsequent contextual analysis was therefore conducted in which coders assigned each pronoun into one of the following categories: (a) *Actual personal pronouns targeting the speaker, the other spouse or the couple*, (b) *Dysfluencies:* pronouns used prior to a repetition (e.g., “*I… I, I* wanted to do that”) or in an interruption of a proposition (e.g., ‘And *I* was, no, …), (c) *Generic*: pronouns referring to a general or universal other (e.g., “*You* always get what *you* pay for”), (d) *Filler*: pronouns used as part of an idiomatic phrase or as a ‘mental comma’ used to fill a speech pause but serve no communicative function (e.g., “*you* know,” “*I* don’t know”), (e) *No code*: pronouns used in references to the speech of a third person (e.g., “Yesterday, Mom said: ‘Now *I* have had enough”’). Only the pronouns that were considered as *actual* personal pronouns (cf. category *a*) were included in the data processing reported below.

### Data Processing

After the contextual analysis, the number of me-words and you-words used by each study participant were summed and divided by the total number of words spoken by this person (”separateness”) and similarly for the number of we-words (“we-ness”). This procedure resulted in four language scores for each partner: “we-ness” expressed in the support-seeker role and in the support-provider role and “separateness” expressed in the support-seeker role and in the support-provider role. Following this procedure, the range of each language score was 0–1. Each transcript was coded by both coders, and the levels of interrater agreement were calculated using the Intraclass Correlation Coefficient (two way random-effects model; absolute agreement) and all of the Intraclass Correlation Coefficients indicated good levels of interrater reliability, both for men (ICC_we-ness_ = 0.99; ICC_separateness_ = 0.95) and women (ICC_we-ness_ = 0.97; ICC_separateness_ = 0.94). Means and standard deviations for the pronoun variables (see **Table 1**) were highly comparable with existing research (Seider et al., 2009; Rohrbaugh et al., 2012).

**Table 1:** Mean proportions and standard deviations for we-ness and separateness.

	Male seeker/Female provider interaction		Female seeker/Male provider interaction	
		
	Men	Women	Men	Women
Separateness	0.074 (*SD* = 0.020)	0.080 (*SD* = 0.022)	0.081 (*SD* = 0.024)	0.076 (*SD* = 0.022)
We-ness	0.008 (*SD* = 0.011)	0.009 (*SD* = 0.010)	0.009 (*SD* = 0.008)	0.008 (*SD* = 0.007)


### Dataset Description

The data discussed in this manuscript have been deposited in Data Archiving and Networked Services (DANS) and are accessible through the following hyperlink http://dx.doi.org/10.5072/dans-2bs-mqh6 under the name ‘A Pronoun Analysis of Flemish Couples’ Support Transactions (Research conducted in Dutch)’. The data contains two files: (1) a.xlsx file containing the pronouns coding-dictionary and the raw data resulting from the pronoun coding by each of the two coders; (2) a.sav file containing all the processed data (demographic data, scale and total scores of the relationship satisfaction questionnaires and mean language scores resulting from the pronoun analysis).

## Author Contributions

All authors listed, have made substantial, direct and intellectual contribution to the work, and approved it for publication.

## Conflict of Interest Statement

The authors declare that the research was conducted in the absence of any commercial or financial relationships that could be construed as a potential conflict of interest.
